# Supplementary figures and images for: Clinical Relevant Immunosuppressive Drugs Differentially Modulate Axonal Outgrowth from Human Stem Cell–Derived Neurons
Source: bioRxiv. 2026 Jul 2:2026.06.29.735084. Preprint. [Version 1] doi: 10.64898/2026.06.29.735084 (PMC13347252; doi:10.64898/2026.06.29.735084)

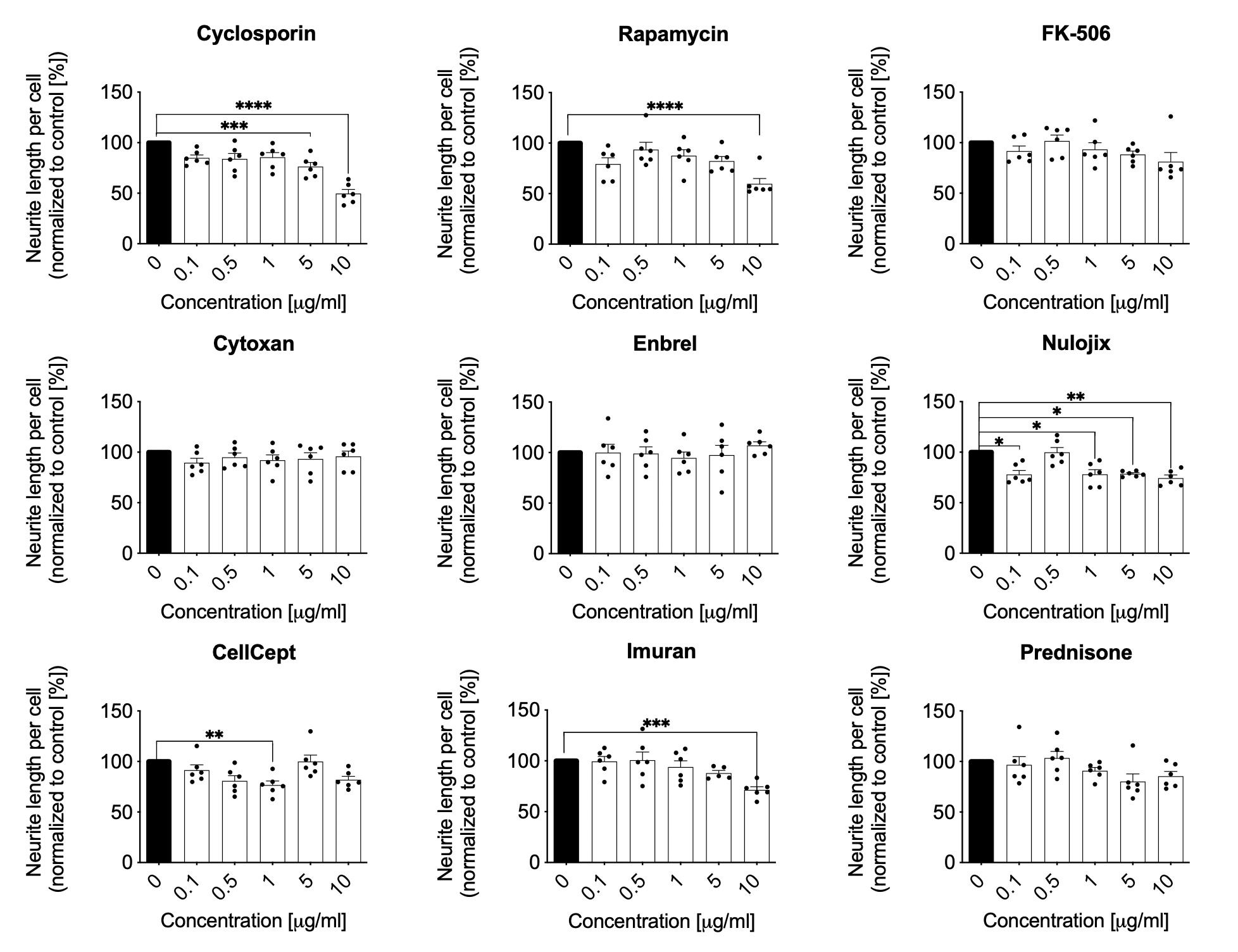

Supplement: Supplement 1 — Figure S1. Axon Growth of hiPSC Derived Neurons is Reduced by Cyclosporine in Vitro (Second patient derived IPSC line) hiPSC derived neurons were plated in 384-well plates and treated 2 hours later with immunosuppressants as indicated (final concentrations range from 0.1 to 10 μg/ml). Shown are total neurite length per cell (Mean+SEM). Cyclosporine showed a significant 50% reduction of neurite growth at 10 μg/ml. Rapamycin showed a significant 40% reduction of neurite growth only at 10 μg/ml (****p<0.0001, one-way ANOVA, with *p<0.05, post-hoc Tukey’s test; n=2 individual experiments, n=3 wells per condition), [file media-1.jpg]

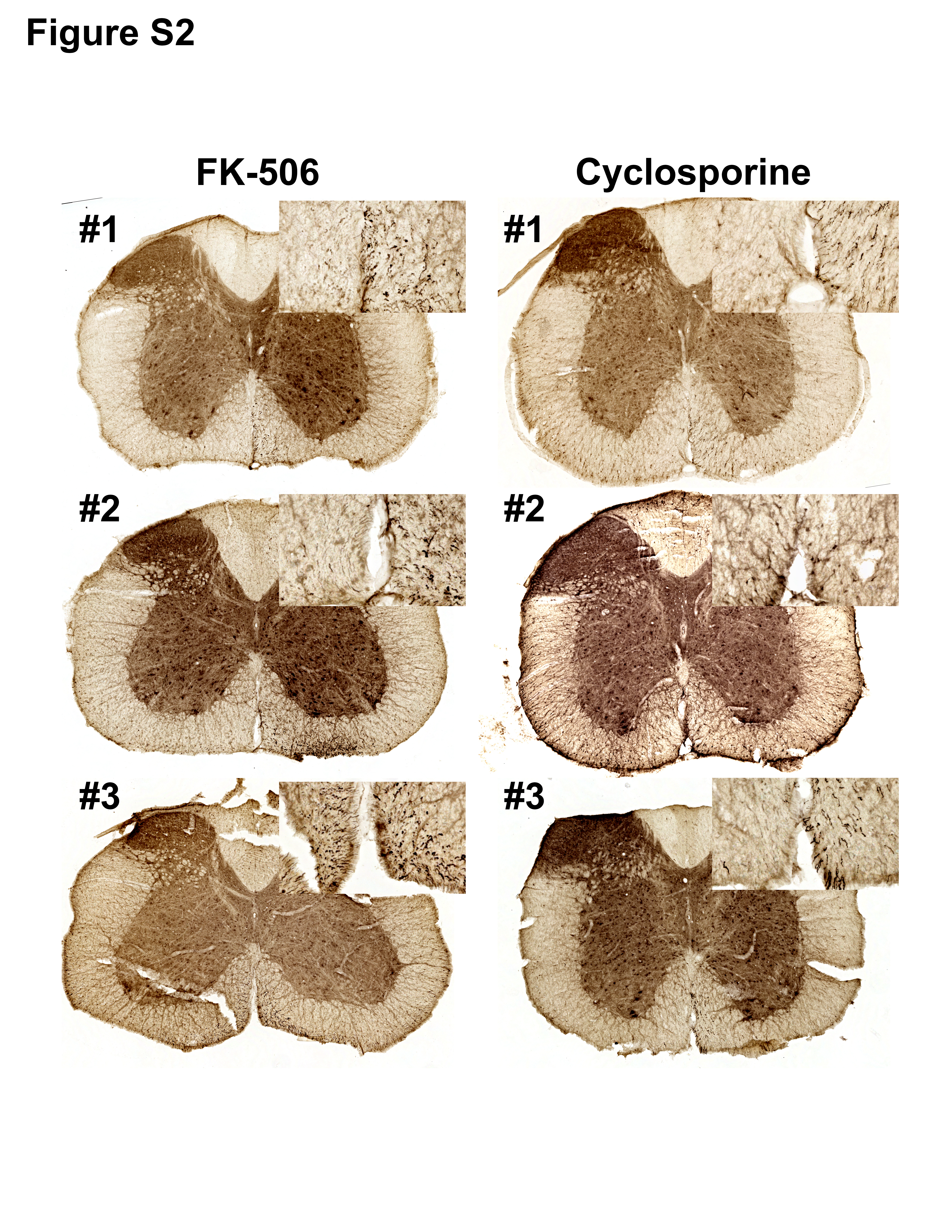

Supplement: Supplement 2 — Figure S2. Human Fetal Spinal Cord Derived NPCs Extend Reduced Numbers of Axons into Spinal Cord White Matter 3 Segments Above Graft Site in Response to Cyclosporine Treatment GFP-expressing fetal human spinal cord-derived NPCs were grafted into sites of C5 intact spinal cord. Mice were either treated daily with FK-506 (left panel) or Cyclosporine (right panel). Two weeks after grafting, transverse sections of spinal cord were immunolabeled for GFP (black). NPC graft-derived axons in transverse sections of mouse spinal cord treated with cyclosporine demonstrates reduced numbers of axons extending into rostral host spinal cord white matter, 3 spinal segments above the graft site. Shown are example images of all biological replicates corresponding to Figure 3A. Scale bar: A, 500μm; magnification, 25μm. [file media-2.jpg]

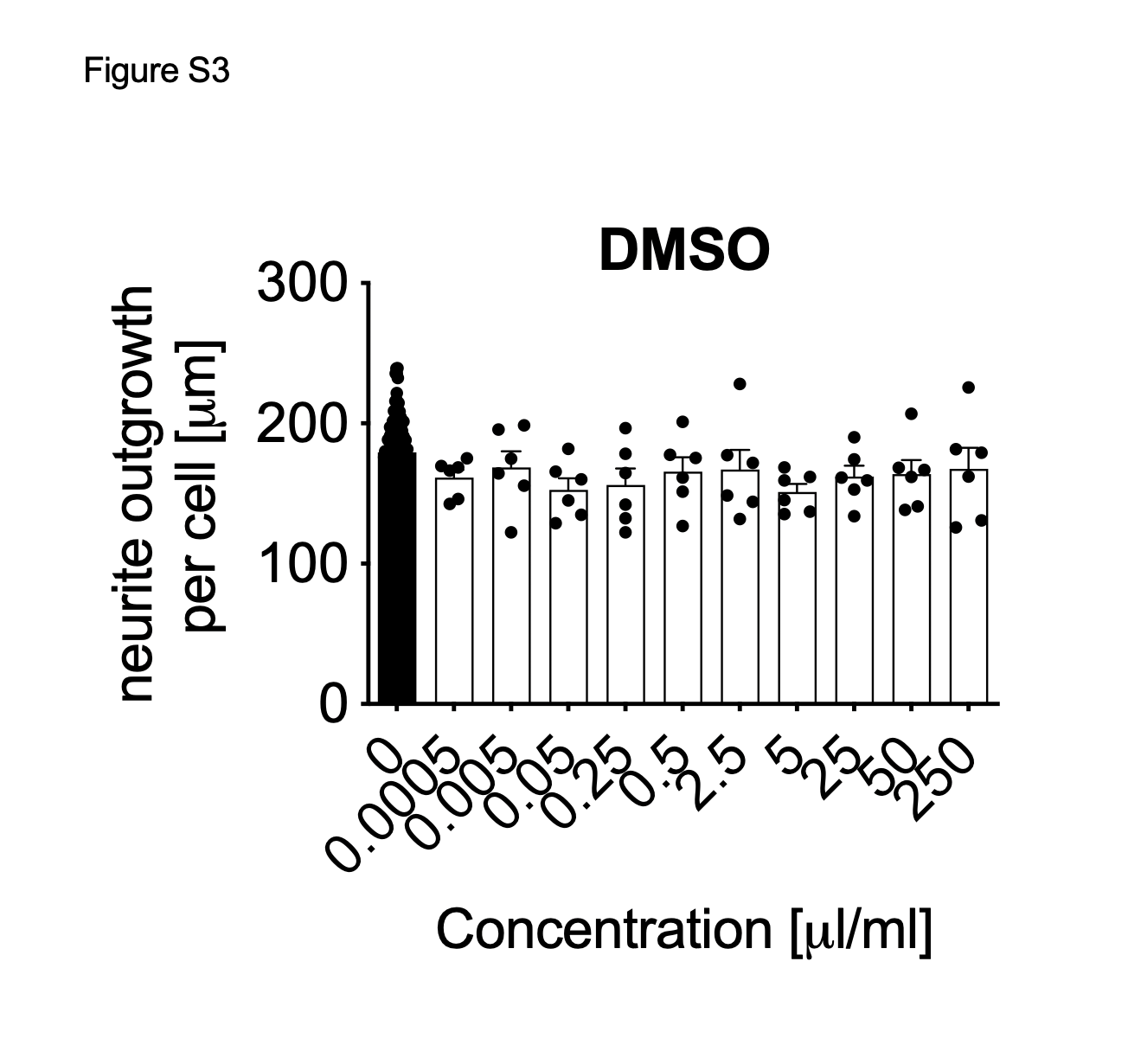

Supplement: Supplement 3 — Figure S3. DMSO Vehicle Does Not Affect Neurite Outgrowth in hiPSC-Derived Neurons. Human iPSC-derived neurons were plated in 384-well plates and treated 2 hours later with DMSO at increasing concentrations (0.00005 to 0.50 μl/ml). Total neurite length per cell was quantified via automated high-content imaging 24 hours later using the MetaXpress neurite outgrowth module. No significant changes in neurite outgrowth were observed across the tested concentrations, indicating that DMSO alone does not impair axonal growth. Data are presented as mean ± SEM (n = 2 independent experiments, n = 3–6 wells per condition). [file media-3.jpg]
